# Exploring the Relationship between Biologics and Postoperative Surgical Morbidity in Ulcerative Colitis: A Review

**DOI:** 10.3390/jcm10040710

**Published:** 2021-02-11

**Authors:** Abel Botelho Quaresma, Fernanda da Silva Barbosa Baraúna, Fábio Vieira Teixeira, Rogério Saad-Hossne, Paulo Gustavo Kotze

**Affiliations:** 1 Department of Colorectal Surgery, Universidade do Oeste de Santa Catarina (UNOESC), Joaçaba 89600-000, Brazil; abel@proctoclinic.com.br; 2 Colorectal Ssurgery Unit, Pontificia Universidade Católica do Paraná (PUCPR), Curitiba 80215-901, Brazil; drafernanda.proctologista@gmail.com; 3 IBD Clinics, Gastrosaúde Clinic, Marília 17509-190, Brazil; fabio@gastrosaude.com; 4 Botucatu Medical School, Paulista State University (UNESP), Botucatu 18618-687, Brazil; rogerio.saad@unesp.br

**Keywords:** ulcerative colitis, antibodies, monoclonal, postoperative complications, infections, necrosis tumor factor-alpha, integrins

## Abstract

Background: With the paradigm shift related to the overspread use of biological agents in the treatment of inflammatory bowel diseases (IBD), several questions emerged from the surgical perspective. Whether the use of biologicals would be associated with higher rates of postoperative complications in ulcerative colitis (UC) patients still remains controversial. Aims: We aimed to analyze the literature, searching for studies that correlated postoperative complications and preoperative exposure to biologics in UC patients, and synthesize these data qualitatively in order to check the possible impact of biologics on postoperative surgical morbidity in this population. Methods: Included studies were identified by electronic search in the PUBMED database according to the PRISMA (Preferred Items of Reports for Systematic Reviews and Meta-Analysis) guidelines. The quality and bias assessments were performed by MINORS (methodological index for non-randomized studies) criteria for non-randomized studies. Results: 608 studies were initially identified, 22 of which were selected for qualitative evaluation. From those, 19 studies (17 retrospective and two prospective) included preoperative anti-TNF. Seven described an increased risk of postoperative complications, and 12 showed no significant increase postoperative morbidity. Only three studies included surgical UC patients with previous use of vedolizumab, two retrospective and one prospective, all with no significant correlation between the drug and an increase in postoperative complication rates. Conclusions: Despite conflicting results, most studies have not shown increased complication rates after abdominal surgical procedures in patients with UC with preoperative exposure to biologics. Further prospective studies are needed to better establish the impact of preoperative biologics and surgical complications in UC.

## 1. Introduction

With the paradigm shift related to the overspread use of biological agents in the treatment of inflammatory bowel diseases (IBD), including a reduction in disease relapse and hospitalizations, several questions emerged from the surgical perspective. An important one is whether the preoperative use of biologics would be associated with higher rates of postoperative complications in ulcerative colitis (UC) patients submitted to colectomy.

Surgery is an important tool in the current therapeutic armamentarium, and is still needed in a significant proportion of patients with UC [1]. Colectomy represents a great challenge to the surgeon due to the severity of the disease and associated disease-related factors, such as malnutrition, infections, anemia, and previous use of corticosteroids [2]. Currently, most patients undergoing an elective colectomy for UC have usually been previously exposed to some type of biological agent [1]. Despite this common scenario, after two decades of using these medications in the treatment of IBD, a cause and effect relationship has not yet been established between a possible increase in surgical complications as a consequence of preoperative exposure to these agents, neither in Crohn’s disease (CD) nor in UC [2,3,4,5,6,7].

Previous publications over the topic are mostly based on retrospective cohort studies with infliximab (IFX) [8,9,10,11,12], and few studies have included patients with other biologics [13,14,15]. These data are based in large heterogeneity, with controversial results [16]. Most of these studies were carried out in patients with CD, followed by patients with IBD (UC and CD in the same analysis). Only one large prospective trial over the topic was presented in an abstract form (PUCCINI trial), which included CD and UC patients [17]. There are few published studies exclusively based on patients with UC that have aimed to analyze the possible impact of biologics in postoperative complications [7,8,13,15,18,19,20].

Our review aimed to analyze the literature, searching for manuscripts that studied the correlation between postoperative complications and preoperative use of biologics exclusively in UC patients submitted to major abdominal surgery. Our qualitative analysis aims to synthesize these data, searching for an answer as to whether biologicals can influence surgical morbidity in this specific population.

## 2. Methods

### 2.1. Search Strategy

This systematic review was carried out in accordance with the recommendations of the Preferred Item Reporting Guidelines for Systematic Reviews and Meta-Analysis (PRISMA) [21]. A complete (unregistered) protocol for systematic reviews was undertaken to achieve the aims of the study. Included studies were identified by electronic search in the Medline database via PubMed (https://www.ncbi.nlm.nih.gov/pubmed/ (accessed on 1 October 2020)). A comprehensive literature search was carried out in October 2020, by title and abstract (TITLE AND ABSTRACT) using the Health Sciences Descriptors developed from the Medical Subject Headings (MeSH terms) of the US National Library of Medicine (NLM) in the following search string:

(((((Postoperative complications) OR Laparoscopy) OR Surgery) OR Colectomy)

AND: (((Inflammatory bowel diseases) OR Colitis, ulcerative)

AND: (((((Biological therapy) OR Tumor necrosis factor alpha), OR Infliximab) OR Adalimumab) OR Integrin alpha4).

NOT: ((((((((Crohn disease), OR Psoriasis), OR Hidradenitis), OR Spondylitis) OR Arthritis) OR Uveitis).

### 2.2. Eligibility and Inclusion and Exclusion Criteria

All terms were searched as keywords when available. The search results were selected for potentially relevant studies by title and abstract, followed by the full-text review of pre-selected publications.

References to relevant publications, such as review articles, have been searched and cross-referenced manually to appropriate additional publications. Publications that met the following criteria were included: studies evaluating postoperative results in patients exclusively with UC who received preoperative treatment with biological agents; published in full in peer-reviewed literature; and only studies originally published in English. Studies carried out in patients with CD and IBD in general (which included CD + UC) were excluded, as well as meta-analyses, editorials, guidelines, erratum, and case reports.

### 2.3. Selection of Studies and Data Collection Process

The citations generated by electronic searches in Pubmed were imported into another specific database. After duplicate citations were removed, the title and abstract of all identified citations were reviewed by two authors (first pass). Full-text publications of potentially relevant citations firstly included were purchased and examined by the same authors (second passage) for inclusion/exclusion, applying the aforementioned eligibility criteria. Any disagreements related to eligibility or interpretation were resolved by a third senior author.

The following data were collected from the selected studies: journal, year of publication, name of the authors, type of article, number of patients, preoperative UC medication, concomitant immunosuppression, duration of treatment with biological agents, postoperative complications (overall complications, infectious complications, surgical site infections, and major postoperative complications), and results with their conclusions.

Data extraction and quality control were performed independently by two reviewers (A.B.Q. and P.G.K.). Disagreements were resolved by a third senior author.

### 2.4. Assessment of Methodological Quality of Studies

As no randomized studies were selected, the methodology was assessed using the methodological index for non-randomized studies (MINORS) [22] on randomized clinical trials and observational studies. The items were scored with 0 (not reported), 1 (reported, but inappropriately), or 2 (reported appropriately). The ideal final score would be 16 for non-comparative studies and 24 for comparative studies. Studies were defined as positive if they demonstrated significant correlation between preoperative biologics and postoperative morbidity, and as negative if they did not.

### 2.5. Ethical Considerations

As a systematic review, this study was waived from review of the medical ethics board.

## 3. Results

**Figure 1:**
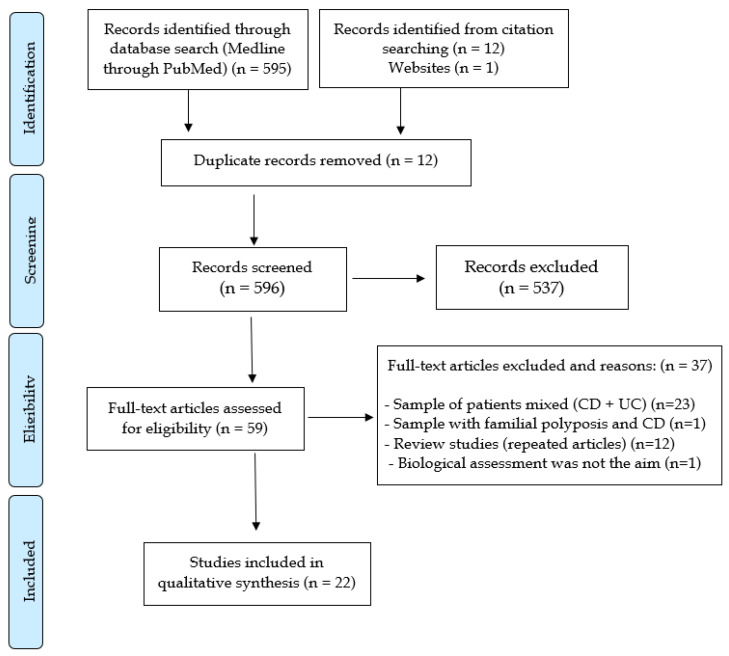
Systematic review PRISMA flow diagram.

Five hundred and ninety-five files were initially selected from the PubMed database. Another 13 relevant articles were retrieved from other sources, with a total of 608 references initially considered for screening. From those, 12 articles were excluded because they were considered duplicates, 537 because they were studies with different outcomes than those proposed for the review, 23 for samples with IBD patients, mixing UC and CD patients, one for containing patients with familial adenomatous polyposis and CD in the same sample, 12 for being reviews based on the same studies already surveyed, and one because of the assessment of biologicals was neither in the objective nor in the conclusions of the study. In the filtering, there were 22 articles left that were used in the qualitative analysis. Figure 1 summarizes the study selection process according to the PRISMA flowchart.

### 3.1. Anti-TNF Therapy

The studies that described a possible impact of anti-TNF agents on increasing postoperative complications exclusively in UC, accompanied by the MINORS quality criteria, are summarized in Table 1. These articles, including mainly patients with IFX, comprised most of the data available in the literature, referring to the relationship between biological agents and postoperative outcomes in UC. The time considered between the last administration of anti-TNF agents before surgery according to most publications was 12 weeks as the ideal cutoff point. This time interval was derived from specific pharmacologic characteristics of IFX [23]. Nineteen studies were published exclusively with patients with UC, 17 being retrospective and only two prospective. Seven studies identified an increased risk of complications with previous use of anti-TNF agents, and 12 showed no significant increased postoperative morbidity related to this class of biologics.

### 3.2. Positive Studies

The seven studies that demonstrated a possible impact of anti-TNF agents in increasing postoperative complications in UC are detailed in Table 2. Most of them were based on retrospective samples [9,10,26] and/or with a small number of patients [24,25].

Kulaylat et al. studied a total of 2476 patients; 950 underwent subtotal colectomy or total colectomy, 354 underwent total proctocolectomy with ileostomy, and 1172 underwent restorative proctocolectomy with ileal-pouch anal anastomosis (IPAA). In the univariate analysis, increased postoperative complications were identified among patients in the IPAA cohort who received anti-TNF agents preoperatively versus those who did not (137 (45.2%) vs. 327 (37.6%); *p* = 0.02). The same result was not observed in the colectomy or total proctocolectomy cohorts. An increase in complications was also observed in the multivariate analysis among patients in the IPAA cohort (OR: 1.38; 95% CI, 1.05–1.82) [34].

Monsinjon et al., in a prospective study from 2017, included 65 consecutive patients (32 men, mean age = 35 (17–87) years) operated on for acute severe ulcerative colitis (ASUC) [18]. Postoperative morbidity was described in 19 patients (29%, group A), and was mainly represented by surgical morbidity (*n* = 15), including ileum (*n* = 9), stoma-related complications (*n* = 5), and intra-abdominal abscess (*n* = 4). Patients with morbidity showed two previous episodes of ASUC (26%) more often than those without morbidity (7%, *p* = 0.04). The duration of anti-TNF treatment was longer than two months in group A (67%) compared with group B (14%, *p* = 0.04). There were no significant differences between groups in relation to other preoperative medical treatments and the number of therapeutic lines.

### 3.3. Negative Studies

The 12 studies that showed no association between previous use of anti-TNF agents and increased rates of postoperative complications are detailed in Table 3. Ferrante et al. retrospectively studied 141 patients with UC who underwent colectomy comparing two groups of patients: 22 patients who received IFX within 12 weeks prior to colectomy and 119 patients who did not [7]. Short-term (30 days) infectious complications, consisting of anastomotic leaks, pelvic abscesses, wound infections, and non-surgical site infections, were increased in patients using corticosteroids (OR 5.19 (95% CI: 1.72–15.66), *p* = 0.003) and/or required ileostomy (OR 6.45 (95% CI: 2.12–19.64), *p* = 0.001) in the postoperative period. In the group that used IFX preoperatively, there was no association with an increased risk of short-term postoperative infectious complications [7].

Uchino et al. prospectively studied 196 patients with UC who underwent laparotomy between January 2010 and September 2012 [19]. The possible factors related to complications were analyzed to identify significant predictors. Twenty-two patients used IFX before surgery. The overall incidence of surgical site infections (SSIs) was 47/196 (24.0%). The incidence of infections, including SSI and other infections, was 69/196 (35.2%). In the multivariate analysis, the national hospital infection surveillance risk index (NNIS) ≥ 2 (*p* < 0.01) and a preoperative prednisolone dose ≥ 0.2 mg/kg/day (*p* = 0.01) were identified as independent risk factors for overall superficial and deep SSI. The NNIS risk index ≥ 2 (*p* < 0.01) and the duration since the onset of UC ≥ 6.3 years (*p* = 0.045) were identified as independent risk factors for superficial SSI; contaminated wound class (*p* <0.01), preoperative hospital stay ≥ 6 days (*p* = 0.048), severe/fulminant disease activity (*p* = 0.04), and pancolitis (*p* = 0, 02) were identified as independent risk factors for deep SSI; and contaminated wounds (*p* < 0.01), severe/fulminant disease activity (*p* = 0.02), and age at surgery ≥ 43 years (*p* = 0.047) were identified as independent risk factors for overall infectious complications. The authors concluded that previous use of IFX was not associated with infectious complications after surgery in patients with UC.

Ward et al., in a large multicenter retrospective study, described the outcomes of 6225 patients undergoing subtotal colectomy, 753 of whom received anti-TNF therapy up to 12 weeks before surgery [20]. From these patients, 418 were in an even shorter period than four weeks. Logistic regression with overall complications as a variable result did not show a significant association between anti-TNF therapy and increased morbidity. Colectomy performed on an urgent basis and smoking were associated with more complications. The authors concluded that there was no association between preoperative anti-TNF therapy and postoperative complications in patients with UC who underwent subtotal colectomy.

### 3.4. Vedolizumab (VDZ)

Three studies that aimed to describe the impact of VDZ in postoperative complications in patients with UC were identified (two retrospective and one prospective). The MINORS quality criteria from these studies are described in Table 4. None of them demonstrated a significant correlation between previous use of VDZ and postoperative morbidity (Table 5).

Lightner et al. retrospectively studied all adult patients with UC who underwent an abdominal operation between 2014 and 2016 and received preoperative VDZ. These were compared to patients with UC with previous exposure to anti-TNF agents. Eighty-eight patients received VDZ, and 62 received anti-TNF agents within 12 weeks of surgery. Patients treated with VDZ had more surgical site infections (*p* = 0.047) and ileostomy dehiscence (*p* = 0.047), but there was no difference in the overall rates of surgical infectious complications or deep SSI, hospital readmissions in 30 days, or return to the operating room. In a univariate analysis among patients with UC, preoperative exposure to VDZ was not a significant predictor of SSI (*p* = 0.27), but steroids were considered as a risk factor (*p* = 0.02). The study concluded that preoperative VDZ is safe for patients with UC [13].

In another retrospective study published in the same year, Ferrante et al. [14] reviewed all colectomies between 2006 and 2016 at the Leuven IBD referral center. Postoperative complications were evaluated within 30 days after surgery in 170 patients. Thirty-four patients (20%) received VDZ up to 16 weeks before surgery and 60 (35%) anti-TNFs within eight weeks. Only the construction of an ileal pouch in the first stage of surgery was an independent factor associated with short-term postoperative infections (OR 2.40; 95% CI, 1.18–4.90, *p* = 0.016). Preoperative therapy (including VDZ) did not influence the short-term outcome, neither in the general population nor in the subpopulation of patients with second-stage ileal pouch construction. The authors suggested, however, that postponing the construction of the pouch for a second-stage surgery in patients undergoing biological therapy or moderate to high doses of steroids is a safer strategy.

Kim et al. studied 285 patients between 2007 and 2017 [15]. Patients were treated with VDZ up to 12 weeks before surgery and were allocated into three treatment groups preoperatively based on age, sex, and surgery (restorative proctocolectomy with IPAA and ileostomy vs. total colectomy with ileostomy), with the following groups: (1) VDZ (*n* = 25); (2) anti-TNF agents (*n* = 74); and (3) patients who did not use biologicals (*n* = 54). Early postoperative complications were compared between the groups of patients. There were no significant differences in the overall incidence of postoperative complications between patients treated pre-operatively with VDZ, anti-TNF agents, or without biologics (44% vs. 45% vs. 37%; *p* = 0.67).

## 4. Discussion

Our review described the main characteristics of studies that analyzed postoperative morbidity and its relation to the previous use of biologics exclusively in UC patients submitted to major abdominal surgery. From the 22 included articles, 19 were based on preoperative anti-TNF therapy (mostly IFX) and three in vedolizumab. Most published studies are based on the possible impact of previous use of anti-TNF agents in postoperative complications, and this is due to the fact that these drugs were approved for use globally years before vedolizumab. Consequently, a greater clinical experience with the class of anti-TNF agents (mostly IFX) is more notable in UC management. No specific studies with golimumab or adalimumab were identified. This can be justified by a better positioning of IFX and VDZ compared to other agents in UC therapeutic strategies with biologics, since these two drugs are the more used biologics globally [35].

Despite the different methodologies used in the studies (most of them being single-center or multi-centric retrospective cohorts), the possible influence of anti-TNF agents in the postoperative course of UC is associated with conflicting results. Overall, 7/19 studies demonstrated a possible association between previous anti-TNFs and higher morbidity, and 12/19 studies did not. Different outcomes for each study constitute an important bias in the combination of data. As an example, surgical postoperative complications cannot be compared between patients submitted to IPAA surgery versus a total colectomy with ileostomy. The incidences of complications vary between these two different procedures, as a possible influence of biologics in postoperative outcomes may depend on the specific type of operation and may be more impactful in restorative proctocolectomy and IPAA. This controversy can be even more illustrated with the different published meta-analyses over the topic, which basically included the same retrospective cohort studies, and reached opposite results [6,36,37,38,39,40,41]. The explanation may lie in the different methods of performing meta-analyses, but mainly in the different outcomes that were analyzed in each publication. Some described infectious complications, and others studied anastomotic leaks and overall complications, among other outcomes. Most of these meta-analyses included patients with CD and UC in the same sample and demonstrated a tendency for these drugs not to affect the postoperative outcomes [6,36,39,40,41]. The diversity of the methods from different meta-analyses comprised the main reason why we did not include this type of article in our inclusion criteria.

Few prospective studies have been published to date aiming to determine the relationship between the previous use of anti-TNF agents and postoperative morbidity in UC [18,19]. The PUCCINI study, a prospective multi-centric American trial published in abstract form in 2019, is probably based on the best level of available evidence to date [17]. Its results demonstrated that detectable levels of anti-TNF agents or previous use of these drugs were not associated with increased postoperative morbidity, a similar finding from most retrospective studies over the topic. The results of this study are solid, mainly due to the measurement of serum levels before surgery in included patients. It is noteworthy that PUCCINI included patients with CD (n = 645) and UC (n = 310) in the same cohort. Postoperative morbidity analysis was carried out by the type of procedure, and not by disease. The final publication is awaited for a more detailed analysis of the results.

All studies included in this systematic review could not demonstrate a cause and effect relationship between previous biologics and higher rates of postoperative complications, as they were basically clinical. Precise markers, as preoperative serum levels of biologicals, were only analyzed in a few publications. Lau et al., in a prospective study from the Cedars Sinai group in Los Angeles, concluded that, in 94 patients with UC undergoing colectomy, adverse postoperative outcome rates between patients with undetectable and detectable levels of IFX were similar when stratified according to the specific type of UC surgery [42]. The PUCCINI trial reached an identical conclusion, but more precise data in the UC patients are awaited [17]. These two important publications were not captured in our systematic review, as they included UC and CD patients, not UC patients exclusively. In addition, there is a lack of studies describing tissue penetration of these agents in surgical samples, which could also mean a precise marker of a cause and effect association.

Data with VDZ comes from more recent studies due to the more recent approval of this drug in clinical practice. The mechanism of action of VDZ, being a gut-selective agent that prevents leukocyte trafficking from the endothelium to intestinal layers, could theoretically impact intestinal anastomoses, as leucocytes are needed for adequate healing. Therefore, speculation as to whether VDZ could increase postoperative complications in UC patients is a natural thought. The Mayo Clinic group published between 2017 and 2018 alarming results in surgical patients with IBD with preoperative use of VDZ [43,44]. In the study, which included exclusively patients with UC, these results were not maintained, and no relationship between the drug and worse postoperative outcomes was clearly identified [13]. Most of the studies with VDZ published later described more reassuring results, placing VDZ as a safe therapy in the perioperative period [14,45,46,47,48,49]. Regarding biologics with other mechanism of action (ustekinumab) or small molecules (tofacitinib), there are no publications in the literature regarding their impact in postoperative morbidity in UC patients. Real-world data with new drugs are awaited in the near future, as the experience with these agents continues to grow over time.

There are few guidelines that recommend an ideal speculated washout period between the last dose of anti-TNF and surgery to minimize postoperative complications. The World Gastroenterological Organization (WGO) stated that if elective IBD-related surgery is needed, it should not be postponed due to previous use of anti-TNFs [20]. The European Crohn’s and Colitis Organisation (ECCO) recommends a surgical procedure in three stages in UC patients with previous biologics. However, no precise indications for the length of interruption of the use of these agents in the preoperative period is detailed [1]. This is a subject far from consensus in the literature.

It is noteworthy that different confounding factors can impact the rates of postoperative complications after colectomy in patients with UC. The most important are the previous use of corticosteroids, an impaired nutritional status, and unfavorable systemic conditions, such as anemia [2,50]. Overall, most patients undergoing colectomy for UC are already using biological agents at the moment of surgery, and more than one of these factors may also be additionally present. Additionally, another important issue that was not included in most reviewed studies was the timing of surgical intervention. Disease duration (time from diagnosis until colectomy) surely influences postoperative outcomes, as patients with a longer disease duration may undergo surgery in worse clinical conditions (malnourished, anemic, and using steroids). This represents another drawback of our systematic review. Surgery for UC can be technically demanding. Total abdominal colectomy or restorative proctocolectomy are considered difficult procedures. IPAA is probably the most complicated operation in colorectal surgery, as it is associated to complications even in patients with adenomatous polyposis. One additional important point is that possible pouch complications may impact future pouch function, addressing the importance of the decision for the proper timing of pouch construction. Therefore, if biologics *per se* constitute the main risk factor for increased postoperative morbidity in UC patients, this remains to be more precisely proved.

Our qualitative systematic review is associated with some limitations that must be taken into consideration before interpreting the data. First, it only included studies exclusively in patients with UC, and important data from studies with both diseases may have been left out. Secondly, it presented studies with low levels of MINORS criteria, mostly retrospective, which may also hinder interpretation. Lastly, there was no quantitative methodology with a meta-analysis, as different outcomes from different studies are difficult to be grouped into more general outcomes. Despite these limitations, the fact that we included only publications exclusively in UC patients undergoing surgery may have better defined the target of the review, as more precise groups of patients were analyzed.

## 5. Conclusions

Although most studies have not shown an increase in the rates of postoperative complications after colectomy for UC in patients using biological therapy, controversy on the subject still persists. Most studies comprise previous use of anti-TNF agents, and few studies with vedolizumab were identified.

There is great heterogeneity in studies with anti-TNF agents, with different results, variation in the definition of outcomes, limited sample of patients, and severity of cases. The prospective PUCCINI trial has described with the best level of available evidence that anti-TNF agents are probably safe in the perioperative period, but more details of the study will only be known after its full publication. A cause and effect relationship between previous anti-TNFs and increased postoperative complications was not identified with the use of preoperative serum levels of the agents as biomarkers. Despite few studies exclusively with UC patients, data with VDZ suggest a similar conclusion, that vedolizumab to date is considered a safe therapy before surgery.

Several confounding factors, such as previous use of corticosteroids, malnutrition, and anemia are usually present in UC patients undergoing colectomy with previous biological therapy and can contribute to increased morbidity. If biologics *per se* increase postoperative complication rates in UC, this still needs to be proved.

An individualized multidisciplinary team, including surgeons and gastroenterologists, in association with increased use of three-stage procedures in UC patients, can lead to optimized outcomes and improved safety, as the complex scenario of colectomy includes not only medication, but other factors that need to be considered in decision-making.

## Figures and Tables

**Table 1:** Methodological index for non-randomized studies (MINORS) for the evaluation of non-randomized clinical trials and observational studies included with anti-TNF.

MINORS	Selvasekar 2007 [9]	Mor, 2008 [10]	Kennedy, 2012 [24]	Eshuis, 2013 [25]	Gu. 2013 [26]	Kulaylat, 2017 [8]	Monsinjon, 2017 [18]	Schluender, 2007 [12]	Ferrante, 2009 [7]	Coquet-Reinier, 2010 [27]	De Silva, 2011 [28]	Gainsbury, 2011 [29]	Bregnbak, 2012 [30]	Schaufler, 2012 [31]	Hicks, 2013 [32]	Uchino, 2013 [19]	Nelson, 2014 [33]	Feuerstein, 2015 [11]	Ward, 2017 [20]
1. A clearly stated aim	2	2	2	2	2	2	2	2	2	2	2	2	2	2	2	2	2	2	2
2. Inclusion of consecutive patients	2	2	1	2	1	1	2	1	2	1	1	1	1	1	1	2	1	1	2
3. Prospective collection of data	1	1	1	1	1	1	2	1	1	1	1	1	1	1	1	2	1	1	1
4. Endpoints appropriate to the aim the study	2	2	2	2	2	2	2	2	2	2	2	2	2	2	2	2	2	2	2
5. Unbiased assessment of the study endpoint	0	0	0	0	0	0	0	0	0	0	0	0	0	0	0	0	0	0	0
6. Follow-up period appropriate to the aim the study	2	2	2	2	2	2	2	2	2	2	2	2	2	2	2	2	2	2	2
7. Loss to follow-up less than 5%	0	0	0	0	0	0	0	0	0	0	0	0	0	0	0	0	0	1	0
8. Prospective calculation of the study size	0	0	0	0	0	0	0	0	0	0	0	0	0	0	0	0	0	0	0
**Additional criteria in the case of comparative study**																			
9. An adequate control group	2	2	2	2	2	0	1	1	2	2	1	2	2	-	0	2	1	1	2
10. Contemporary groups	2	2	2	2	2	2	1	1	2	2	2	1	1	-	2	2	2	2	1
11. Baseline equivalence of groups	1	1	1	1	1	1	1	1	1	1	1	1	1	-	1	1	1	1	1
12. Adequate statistical analyses	2	2	2	2	2	2	2	2	2	2	2	2	2	-	2	2	2	2	2
Total	**16**	**16**	**15**	**16**	**15**	**13**	**16**	**13**	**16**	**15**	**14**	**14**	**14**	**8**	**13**	**17**	**14**	**15**	**15**

The items are scored 0 (not reported), 1 (reported, but inadequate), or 2 (reported and adequate). The global ideal score was 16 for non-comparative studies and 24 for comparative studies. Bold: total scores for each study.

**Table 2:** Studies that demonstrated an increase in rates of postoperative complications in patients with UC (positive studies).

Author	Journal	Year	Type of Study	Biological Agent	Cohort/ Treated	Main Outcomes Analyzed
Selvasekar et al. [9]	J Am Coll Surg	2007	Retrospective single-center	IFX	301/47	Short-term postoperative complications
Mor et al. [10]	Dis Colon Rectum	2008	Retrospective single-center	IFX	523/85	Overall postoperative complications
Kennedy et al. [33]	J Pediatr Surg.	2012	Retrospective single-center	IFX	11/38	Complications after IPAA in pediatric patients
Eshuis et al. [25]	J Crohn’s Colitis	2013	Retrospective single-center	IFX	72/38	Overall postoperative complications
Gu et al. [26]	Dis Colon Rectum	2013	Retrospective single-center	Anti-TNF	181/25	Overall infectious postoperative complications
Kulaylat et al. [8]	JAMA Surg.	2017	Retrospective Single-center	Anti-TNF	2476/650	Overall postoperative complications
Monsinjon et al. [18]	Int J Colorectal Dis.	2017	Prospective	Anti-TNF	65/65	Postoperative morbidity in laparoscopic patientes

**Table 3:** Studies that did not show increased morbidity related to the use of preoperative anti-TNF agents (negative studies).

Author	Journal	Year	Type of Study	Cohort/ Treated	Biological	Main Outcomes Analyzed
Schluender et al. [12]	Dis Colon Rectum	2007	Retrospective single-center	151/17	IFX	Postoperative medical and surgical complications
Ferrante et al. [7]	Inflamm Bowel Dis	2009	Retrospective single-center	141/22	IFX	Short-term infectious complications
Coquet-Reinier et al. [27]	Surg Endosc.	2010	Retrospective single-center	26/13	IFX	IPAA-related complications
De Silva et al. [28]	Clin Gastroenterol Hepatol	2011	Retrospective single-center	666/58	IFX	Severe postoperative complications including in-hospital mortality
Gainsbury et al. [29]	J Gastrointest Surg	2011	Retrospective single-center	81/29	IFX	Short-term complications
Bregnbak et al. [30]	J Crohn’s Colitis	2012	Retrospective single-center	71/20	IFX	Short-term complications
Schaufler et al. [31]	J Pediatr Gastroenterol Nutr	2012	Retrospective single-center	51/33	IFX	Preoperative complications in children
Hicks et al. [32]	Am J Surg	2013	Retrospective single-center	179/43	IFX	Overall complications in patients undergoing urgent versus elective surgery for UC
Uchino et al. [19]	Int J Colorectal Dis	2013	Prospective single-center	196/22	IFX	Identify possible significant predictors related to overall complications
Nelson et al. [33]	Inflamm Bowel Dis	2014	Retrospective single-center	78/28	IFX	Infectious, non-infectious, and overall early complications
Feuerstein et al. [11]	Inflamm Bowel Dis	2015	Retrospective single-center	209/24	Anti-TNF	Readmissions rates
Ward, ST et al. [20]	Colorectal Dis	2017	Retrospective multicenter	6225/753	Anti-TNF	Overall postoperative complications

**Table 4:** Methodological index for non-randomized studies (MINORS) for the evaluation of non-randomized clinical trials and observational studies included with vedolizumab.

MINORS	Lightner, 2017 [13]	Ferrante, 2017 [14]	Kim, 2020 [15]
1.A clearly stated aim	2	2	2
2.Inclusion of consecutive patients	2	2	2
3.Prospective collection of data	1	1	2
4.Endpoints appropriate to the aim the study	2	2	2
5.Unbiased assessment of the study endpoint	0	0	0
6.Follow-up period appropriate to the aim the study	2	2	2
7. Loss to follow-up less than 5%	0	0	1
8.Prospective calculation of the study size	0	0	0
**Additional criteria in the case of comparative study**			
9.An adequate control group	2	2	2
10.Contemporary groups	2	2	2
11.Baseline equivalence of groups	1	1	2
12.Adequate statistical analyses	2	2	2
**Total**	**16**	**16**	**19**

The items are scored 0 (not reported), 1 (reported, but inadequate), or 2 (reported and adequate). The global ideal score was 16 for non-comparative studies and 24 for comparative studies. Bold: total scores for each study.

**Table 5:** Studies that correlated preoperative use of VDZ with postoperative morbidity.

Author	Journal	Year	Type of Study	Biological	Cohort / Treated	Main Outcomes Analyzed
Lightner et al. [13]	J Crohn’s Colitis	2017	Retrospective single-center	VDZ Anti-TNF	*N* = 170 62 anti-TNF 88 VDZ	Overall postoperative infectious complications
Ferrante et al. [14]	J Crohn’s Colitis	2017	Retrospective single0center	VDZ, Anti-TNF	*N* = 170 60 anti-TNF 34 VDZ	Overall postoperative complications
Kim et al. [15]	BMC Surgery	2020	Multicenter prospective	VDZ, Anti-TNF	*N* = 153 25 VDZ 74 Anti-TNF 54 in biologics	Overall postoperative complications
